# Mitochondrial Peroxiredoxin-IIF (PRXIIF) Activity and Function during Seed Aging

**DOI:** 10.3390/antiox11071226

**Published:** 2022-06-23

**Authors:** Ewelina A. Klupczyńska, Karl-Josef Dietz, Arleta Małecka, Ewelina Ratajczak

**Affiliations:** 1 Institute of Dendrology, Polish Academy of Sciences, 62-035 Kórnik, Poland; 2 Biochemistry and Physiology of Plants, Faculty of Biology, Bielefeld University, 33615 Bielefeld, Germany; karl-josef.dietz@uni-bielefeld.de; 3 Laboratory of Biotechnology, Institute of Molecular Biology and Biotechnology, Adam Mickiewicz University, 61-614 Poznan, Poland; arleta.malecka@wco.pl; 4 The Cancer Epidemiology and Prevention Unit, Garbary 15 Street, 61-866 Poznan, Poland

**Keywords:** seed aging, peroxiredoxin-IIF, thioredoxin, redox regulation

## Abstract

Mitochondria play a major role in energy metabolism, particularly in cell respiration, cellular metabolism, and signal transduction, and are also involved in other processes, such as cell signaling, cell cycle control, cell growth, differentiation and apoptosis. Programmed cell death is associated with the production of reactive oxygen species (ROS) and a concomitant decrease in antioxidant capacity, which, in turn, determines the aging of living organisms and organs and thus also seeds. During the aging process, cell redox homeostasis is disrupted, and these changes decrease the viability of stored seeds. Mitochondrial peroxiredoxin-IIF (PRXIIF), a thiol peroxidase, has a significant role in protecting the cell and sensing oxidative stress that occurs during the disturbance of redox homeostasis. Thioredoxins (TRXs), which function as redox transmitters and switch protein function in mitochondria, can regulate respiratory metabolism. TRXs serve as electron donors to PRXIIF, as shown in Arabidopsis. In contrast, sulfiredoxin (SRX) can regenerate mitochondrial PRXIIF once hyperoxidized to sulfinic acid. To protect against oxidative stress, another type of thiol peroxidases, glutathione peroxidase-like protein (GPXL), is important and receives electrons from the TRX system. They remove peroxides produced in the mitochondrial matrix. However, the TRX/PRX and TRX/GPXL systems are not well understood in mitochondria. Knowledge of both systems is important because these systems play an important role in stress sensing, response and acclimation, including redox imbalance and generation of ROS and reactive nitrogen species (RNS). The TRX/PRX and TRX/GPXL systems are important for maintaining cellular ROS homeostasis and maintaining redox homeostasis under stress conditions. This minireview focuses on the functions of PRXIIF discovered in plant cells approximately 20 years ago and addresses the question of how PRXIIF affects seed viability maintenance and aging. Increasing evidence suggests that the mitochondrial PRXIIF plays a major role in metabolic processes in seeds, which was not previously known.

## 1. Introduction

Peroxiredoxins (PRXs, EC 1.11.1.15) are thiol-dependent peroxidases found in all organisms. They were first described in *Salmonella typhimurium* as alkyl hydroperoxide reductase C (AhpC) [1]. PRX are ubiquitous proteins belonging to a family of antioxidant enzymes that scavenge reactive oxygen species (ROS) with peroxyl moieties, i.e., compounds with the general structure R-O-O-H, where R either is hydrogen (hydrogen peroxide, H_2_O_2_), a shorter (e.g., butylhydroperoxide: (CH_3_)_3_COOH) or bulky alkyl residue (cumenehydroperoxide) or NO (peroxynitrite: ONOO^−^) [2].

PRXs are involved in many eukaryotic redox regulatory pathways and affect signal transduction in cells. In animals, PRXs affect cytokine-dependent signaling [3]. Other PRX isoforms provide defense against oxidative damage, and others are involved in signal transduction by controlling the H_2_O_2_ concentration [4]. Thiol oxidation of certain PRXs leads to the formation of macromolecular multimers [5] that adopt molecular chaperone activity. The oxidized form of PRX cannot function as peroxide reductase [6]. Once reductively regenerated, PRXs can reduce H_2_O_2_ to two H_2_O molecules, alkyl hydroperoxides to the corresponding alcohols and ONOO^−^ to nitrite, the latter two with a stoichiometric release of one molecule of H_2_O [2,3].

H_2_O_2_ is a major ROS form in biology generated either via single reduction of O_2_ to superoxide radical (O_2_^−●^), e.g., in the photosynthetic electron transport (PET) or respiratory electron transport (RET) chains, followed by spontaneous or catalyzed dismutation, or directly by two-electron transfer to O_2_ in oxidase reactions such as peroxisomal glycolate oxidase in photorespiration (glycolate + O_2_^−^ → glyoxylate + H_2_O_2_).

**Figure 1:**
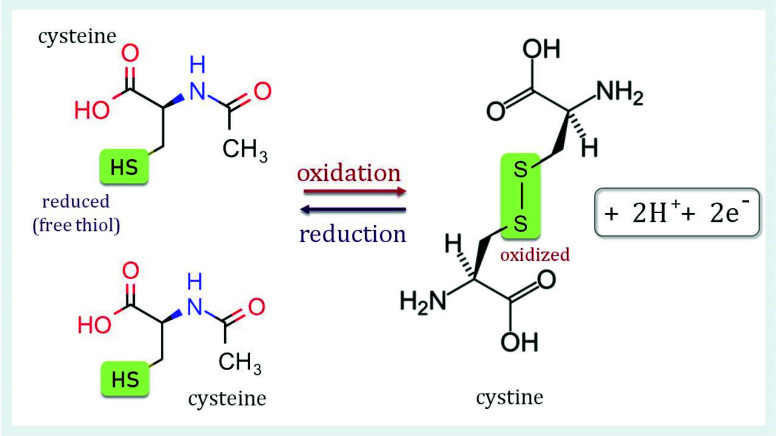
Disulfide bridge formation in cystine. A disulfide bridge can form between two sulfur atoms (-S-S-) of two identical or different thiol compounds. Substances containing such bridges are called disulfides. In proteins, disulfide bonds are formed between the sulfur atoms of two cysteinyl residues yielding cystine. Disulfides stabilize the tertiary structure of proteins. This covalent structural feature is the strongest force in protein structure formation and complements ionic salt bridges, hydrogen bonds and van der Waals forces.

Electron donors for regeneration of PRX, also called redox transmitters, vary among subtypes and biological species [7,8]. All PRXs contain a conserved cysteinyl residue with a deprotonated thiol group at the more N-terminal part of the protein. This peroxidatic cysteinyl residue (C_P_) is the primary H_2_O_2_ binding site. Apart from the subgroup of 1-CysPRXs, a second cysteinyl at the more C-terminal part of the protein functions as resolving thiol, resolving cysteinyl residue (C_R_). It is involved in the next step of the peroxide detoxification of all typical and atypical 2-CysPRX. Using both cysteinyl residues, PRXs maintain their latent catalytic activity by formation of a disulfide bond between C_P_ and C_R_ (Figure 1). The last step involves the conversion of the oxidized protein to its reduced conformation by redox transmitters.

Overall, the catalytic cycle comprises three steps, the first step of peroxide reduction and C_P_ oxidation, the second step of disulfide formation and the third step of disulfide reduction by a suitable electron donor. It is apparent that the function of PRXs decisively depends on reversible thiol (-(SH)_2_)/disulfide (-S-S-) exchange reactions, which are thermodynamically and kinetically linked to the redox potentials of the glutathione pool and the NADPH/NADP^+^-redox couple or, in the chloroplast PET, of the ferredoxin system [9].

## 2. Seed Aging and Changes in Redox State

The viability and aging of seeds depend on genetically determined properties on the one hand and physicochemical properties like their water contents, the tissue oxygen levels and the storage temperature on the other hand. These parameters affect the metabolic and biophysical properties of the seed. Based on these properties, seeds are divided into three categories: orthodox seeds that tolerate moisture content <7% and storage at −10 °C, recalcitrant seeds that are sensitive to drying and cannot tolerate moisture content <7%, and intermediate seeds with properties between both categories.

Aging processes reduce seed viability during storage and lead to deterioration of quality. Seed aging is manifested at the levels of plant development and physiology, ranging from morphological changes to genetic damage. The profound disturbance persists during delayed, and in extreme cases, suspended germination of aged seeds. The primary hallmarks of seed aging seem to be ROS formation that initiates oxidative and peroxidatic processes. ROS react with unsaturated fatty acids and proteins, also in cell membranes that lose their barrier function. Ion leakage is another common hallmark of seed aging. Other indicators of seed aging are changes in the composition of the proteome, increased protein degradation, and changes in the activity and regulation of enzymes [10].

During seed aging, ROS and redox changes alter gene expression that in cases of severe redox disequilibria initiate programmed cell death (PCD). As in other plant cells and tissues, redox-dependent activation of the voltage-dependent anion-selective channel (VDAC) may allow for release of cytochrome c from the mitochondrial intermembrane space and induce PCD [11]. Accompanied DNA fragmentation leads to the ladder structure of DNA, and contraction of the cytoplasm. Two proteases, RD21 and papain, are present in plant cells, the action of which is similar to the action of caspase in animal cells [12]. According to the “free radical theory” proposed by Harman (1956), the accumulation of free radical damage is the main cause of aging in living organisms; therefore, prolonged or improper storage is considered to be the main cause of seed degradation [13].

**Figure 2:**
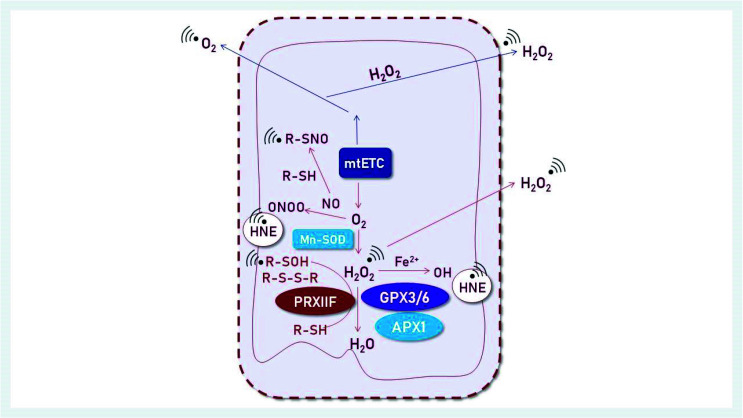
Mitochondrial ROS defense system. Redox metabolism is associated with the generation of reactive oxygen and nitrogen species (ROS, RNS). The mitochondrial electron transport chain (mtETC) is a major ROS generator. The process of ROS elimination and redox signal integration involves mitochondrial antioxidant enzymes such as manganese superoxide dismutase (MnSOD) and ascorbate-dependent peroxidase (APX). MnSOD, APX and thiol peroxidases of the PRX and glutathione peroxidase-like type (GPXL) detoxify O_2_^−●^ and H_2_O_2_. Thiol peroxidases also function as redox-sensitive proteins. TRX and GRX together with glutathione are involved in ROS elimination and redox signal integration in mitochondria [14]. HNE–4-hydroxynonenal (C_9_H_16_O_2_) is produced by lipid peroxidation, and plays an important role in cell signal transduction.

Mitochondria are vulnerable to ROS damage, because they are the production sites of ROS in the respiratory chain (Figure 2). Reduced activity of mitochondrial respiration enzymes was also observed, which may be related to damage to the mitochondrial membranes. Moreover, phospholipid substrates, which build the mitochondrial membranes, may be peroxidized by oxygen radical attacks.

As shown in previous studies, ROS, including H_2_O_2_, formed in mitochondria contribute to the acceleration of processes associated with seed aging [15]. Aging cells with an increasing proportion of dysfunctional mitochondria are less functional than non-aging cells and may die as a result of metabolic disorder. Mitochondria have an efficient antioxidant system that counteracts accumulation of ROS to protect cells from aging [15].

Nevertheless, our understanding of the exact role of mitochondria in seed aging is still incomplete. Thus, it is timely to address the role of mitochondrial dysfunction in seed aging. The question is: how mitochondrial dysfunction connects with seed aging during storage [15]? The question arises from the fact that ROS levels increase during seed storage because ROS are continuously produced in mitochondria of stored seeds [15]. The likely hypothesis is that both processes mutually interfere and possibly potentiate each other, until the point-of-no-return of irreversible damage is reached and germination ceases.

## 3. Redox Signaling in Mitochondria

### 3.1. ROS Generation

Mitochondria are the energy centers of the heterotrophic cell. Protein complexes of the mitochondrial respiratory electron transport chain (RET) during oxidative phosphorylation are also the most important sites of ROS production [16,17], mainly in complexes I, II and III on the respiratory chain. Superoxide anion radicals (O_2_^−●^) form by single-electron transfer to oxygen as byproduct in the RET. Two O_2_^−●^ spontaneously dismutate to H_2_O_2_ and water or via the enzyme SOD, superoxide dismutase [18]. At pH 7, SOD accelerates the rate constant by a factor of about 1000 [19]. H_2_O_2_ is produced by the two-electron reduction of the oxygen molecule, or by dismutation of 2 O_2_^−●^ mentioned above. Abiotic and biotic stresses stimulate mitochondrial ROS production [20].

Apart from their seemingly negative impact, namely by oxidizing various cell constituents in an uncontrolled and damaging manner, ROS also show positive features. They initiate or modulate cell signal transduction and regulatory networks, e.g., by tuning gene expression, protein synthesis or metabolism, and thereby participate in processes related to environmental acclimation, in defense against pathogens or in plant development, such as lateral root formation and sprouting stimulus [21,22]. How they interact with the cellular network largely depends on the spatial and temporal accumulation, which, in turn, is determined by the rate of ROS production, the activity of the antioxidant systems and the presence of redox sensors and target proteins [23,24,25]. Increased ROS accumulation in subcellular compartments shifts the glutathione redox potential to more positive values, which subsequently alters the activity of proteins, in particular, by *S*-glutathionylation of sensitive thiols in the proteins. By participating in the regulation of nuclear gene transcription, ROS are important candidates as signaling molecules to coordinate mitochondrial biochemistry and nuclear gene expression as retrograde mechanism [26]. However, ROS alone cannot initiate a specific response to developmental and environmental cues. To do so, ROS integrate with other components of cellular signaling pathways, such as RNS, hormones, or intracellular Ca^2+^ and developmental, environmental or circadian predisposition [23,27].

As mentioned before, O_2_^−●^ often is the first ROS type produced by generator systems such as RET. Therefore, the first component of the antioxidant defense system is the family of SODs, which occur in three isoforms distinguished by their metal cofactor: FeSOD, CuZnSOD and MnSOD. MnSOD is characteristic for mitochondria and peroxisomes. Reduced activity of MnSOD disturbs mitochondrial redox balance and affects plant growth [28]. Interestingly, transcripts encoding MnSOD are often not as responsive to abiotic stresses as CuZnSOD [29,30].

ROS metabolism and redox regulatory processes initiated by ROS in different cellular compartments are interrelated and involved in optimizing cellular functions through different pathways. Subcellular compartments, such as chloroplasts, mitochondria, peroxisomes, cytosol or apoplast, establish and control their own ROS homeostasis, but gradients in redox state in these compartments generate a stimulus-specific ROS signal [22]. Via aquaporins, the H_2_O_2_ signal is transmitted to the cytosol.

Controlling ROS production and avoiding ROS accumulation by removal of their excess establish adequate cellular redox conditions that are required for regular plant development and physiology. Up to now, experimental methods are unavailable that quantify absolute steady-state ROS concentrations in vivo. Cell imaging with H_2_O_2_-sensitive probes only monitor relative changes [31]. Mathematical modelling based on kinetic constants and comparison with determined ex vivo redox state of 2-CysPRX gave an estimate of resting H_2_O_2_ concentrations in the range of 30 nM [32].

ROS at low concentrations are needed as oxidants within the redox regulatory thiol network [23]. Concentrations below the resting level may negatively interfere with cellular functions and affect the immune response. For plant growth and development to take place properly, cells must adjust adequate ROS concentrations so that they are neither too high nor too low and maintain redox homeostasis.

These examples show that, within a certain low concentration range, ROS function as relay molecules in signaling networks, which are closely related to the maintenance of proper redox responses. Homeostasis of the redox state affects almost every aspect of plant biology, such as cell proliferation, metabolism, growth and regulation of plant development, defense responses, and cell death [33].

### 3.2. The Role of Mitochondria in Cell Redox Homeostasis

The plant mitochondrial RET is a branched electron transport pathway of canonical and alternative inputs and terminal oxidases. Flexibility in electron:proton:ATP coupling is required depending on environmental conditions, e.g., by use of alternative NAD(P)H dehydrogenase (NDH type II) and alternative oxidase (AOX). The conditional steering of the alternative pathways plays a role in regulating the cellular redox state and preventing excessive reduction of the NAD(P)H systems and the mitochondrial electron transport chain with concomitantly enhanced production of excess ROS [34,35]. The contribution of plant mitochondria to ROS production is usually low due to the presence of AOX, catalyzing the reduction of O_2_ by ubiquinol and avoiding the proton pumping and phosphorylation of ADP by F-ATP synthase (an ATPase/synthase found in mitochondrial inner membranes in oxidative phosphorylation, where it is known as Complex V) [36].

**Figure 3:**
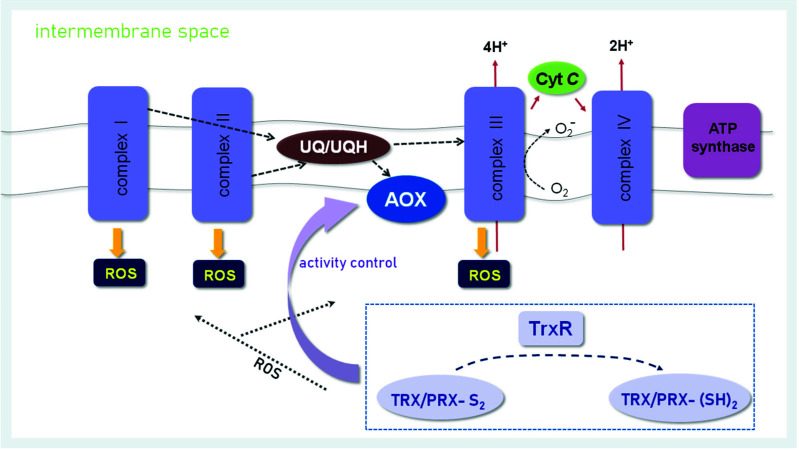
During long-term seed storage, mitochondrial structure is damaged in seeds due to increased ROS levels, oxidative damage to membranes, loss of enzyme activities and reduced activity of the antioxidant system [41]. We hypothesize that, during seed storage, under the influence of ROS, changes in metabolic activity and respiratory electron transport chain initiate the switch to the alternative respiratory pathway. The switch to ubiquinone-linked AOX for electron transport is regulated by mitochondrial TRX, changes in metabolite concentrations and by transcriptional control. During long-term seed storage, a decrease in the activity of systems regulating the redox state of cells is observed. The level of thiol group-containing proteins, i.e., TRXs and PRXs, mainly decreases [15], i.e., proteins that are actively involved in regulating the level of ROS and in modulating redox signaling. The decrease in the activity of TRX and PRX, on the one hand, likely affects the decrease in AOX activity and disrupts the phosphorylating electron transport and energy production needed by the cell to function properly. On the other hand, the decrease in the activity of TRX and PRX will lower the antioxidant defense, increase the ROS level and enhance mitochondrial dysfunction. The sum of all these events accelerates the aging process and reduces seed viability.

The role of AOX in respiration is evident under environmental stress conditions and during plant development [37]. AOXs support respiratory metabolism when the efficiency of complex IV or complex III of the phosphorylating pathway is low. AOXs receive electrons immediately from ubiquinol, preventing electron transfer to complex III and IV and over-reduction of RET, and thus lowers the membrane potential and counteracts the production of ROS in complex III [38]. Its involvement in respiration may protect against oxidative stress. Because AOX can be activated during both abiotic and biotic stress—it is active independently of the stress but its involvement in O_2_ uptake may increase during the stress—it is referred to as a survival protein that promotes cellular redox homeostasis [39]. AOX isogenes are encoded by the nuclear genome, and the protein itself is associated with the inner mitochondrial membrane; therefore, its expression depends on the activity of RET and undergoes mitochondrial feedback regulation [40] (Figure 3).

AOX genes exist in subfamilies named AOX1 and AOX2. AOX2 expression is greater in reproductive tissues and seeds. AOX transcripts are induced primarily by stress stimuli; therefore, exogenous ROS are one of the factors that activate AOX expression. AOX has two highly conserved Cys residues in its structure, and TRXs regulate the activity of this enzyme in vitro by reducing the Cys groups. In addition, pyruvate and different tricarboxylic acid cycle (TCA cycle) intermediates decisively participate in regulating AOX activity [42,43]. Recently, the thiol redox state and activity of AOX was scrutinized in *trx-o1* knock *Arabidopsis thaliana* L. lines after transfer from normal growth light to high light [44]. Under conditions of excess excitation energy, AOX is activated to dissipate reducing power from the chloroplast. This work that also relied on metabolome and metabolite flux analyses established a complex interaction between TRX-dependent control of AOX activity and state of the TCA cycle. The AOX thiol redox state was unaltered, but still the AOX and TCA cycle activities were upregulated in response to high light, indicating enhanced energy dissipation. The results point to hitherto unrecognized mechanisms of regulation of metabolic pathways, energetic coupling and dissipation of excess energy. 

TRXs not only are involved in the adjustment of AOX thiols, but are important in regulating the redox state of mitochondria in a broader context [34,43,45,46]. The presence of TRX in plant mitochondria was demonstrated by Laloi et al. [47] in Arabidopsis. Mitochondrial TRXs were classified as the TRX-o type (AtTRX-o1). Also, h-type TRX was localized in mitochondria of poplar [48]. The latter work identified PtTRX-h2 in mitochondria and suggested that TRX-h2 might also be able to reduce AOX disulfides.

TRXs catalyze the dithiol–disulfide transition. Many mitochondrial proteins were identified as interactions partners of TRXs, e.g., isocitrate dehydrogenase, malate dehydrogenase, succinate dehydrogenase and MnSOD [45,47]. Thiol-dependent redox processes play a role in regulation and catalysis. TRX-o donates electrons to PRXIIF [49] and presumably to glutathione peroxidase-like proteins such as the mitochondrial GPXL6 [50]. Both thiol peroxidases reduce H_2_O_2_ and other alkylhydroperoxides to H_2_O or corresponding alcohols. Glutaredoxins (GRXs) are also important in redox signaling by linking protein thiol status to the redox status of the glutathione pool and/or ROS production [51]. In mitochondria, the presumably only GRX is the monothiol GRXS15, which functions in Fe-S cluster assembly [52]. Plants devoid of this GRXS15 are embryo-lethal. RET contains 12 FeS-proteins, and the mitochondrion at least 26 [4Fe-4S] proteins. GRXS15 deficiency affects enzymes and metabolites of the citric acid cycle and specifically impedes lipoylation; however, it did not affect the mitochondrial ROS state [52,53].

Plant mitochondria also produce nitric oxide (NO) by reducing nitrite, although the site of NO formation remains uncertain [54,55]. The reaction product of superoxide and NO is toxic to animal cells at micromolar concentrations but has no detectable effect on plant cells [54] (Figure 2).

### 3.3. Peroxide Detoxification by Peroxiredoxins

PRXs share a common catalytic mechanism in which, by the reduction of peroxide, the Cys catalytic active site is oxidized to sulfenic acid, which then forms a disulfide bond with the resolving Cys. Prior to the next catalytic cycle, the disulfide must be reduced by a redox transmitter, e.g., by suitable thioredoxins in the NADPH → NTR → TRX → PRX-system [56] or by glutathione/glutaredoxins in the NADPH → glutathione reductase → glutathione → GRX → PRX-system as in the case of the chloroplast PRXIIE [57]. Plant mitochondria contain a single atypical PRX that was named PRXIIF [58].

The PRXIIF catalytic cycle consists of three steps. In step 1, the nucleophilic attack on the peroxide by the conserved peroxidatic cysteinyl thiol (Cys-S PH), which is oxidized to sulfenic acid (Cys-SP OH). In step 2, the disulfide is formed by attack of the sulfenic acid derivative by the free thiol of the resolving cysteinyl residue (Cys-S RH) to release water. In step 3, the thiol form is regenerated mainly by mitochondrial TRX [49,59]. Thus, the catalytic cycle of PRXIIF is coupled to the NADPH → NTR → TRX-system.

PRXs exert signaling functions, e.g., by acting as cell cycle regulators [60] or by regulating metabolism [61]. These functions depend on certain molecular mechanisms such as (i) formation of intermolecular disulfide bonds with partner proteins, (ii) controlling the redox state of redox transmitters that, in turn, switch target proteins, (iii) functioning as chaperone proteins or (iv) interacting in a redox-state-dependent manner with proteins, as shown by interactome proteomics, e.g., for the chloroplast 2-CysPRX [5,59].

Analysis of the daily dynamics of the PRX redox state revealed that the occurrence and disappearance of the sulfonic acid (= hyperoxidized) form of PRX (including PRXIIF) displays a circadian rhythm and may serve as a daily rhythmic biomarker [62]. This feature was also observed in nucleus-free erythrocytes and in cells with blocked transcription. Therefore, the circadian rhythm of PRX hyperoxidation is independent on transcription, and was suggested to serve in an evolutionarily ancient translation–transcription feedback loop [62]. In cells with functional transcription, it is likely that there is a complex interaction and crosstalk between transcription-dependent processes and transcription-independent oscillations. 

In plants, the circadian clock functions as an endogenous 24 h oscillator, regulating many critical biological processes. The circadian clock also influences and partly controls seed germination [63]. The circadian clock is temperature-adjusted and entrained by environmental cues, which helps maintain a 24 h rhythm over a wide range of physiological conditions. Circadian oscillations in cytoplasmic redox parameters, including high amplitude oxidation–reduction of PRXs [62], raise the question of whether a plant mitochondrial antioxidant such as PRXIIF can effectively serve in circadian clock-dependent activities. It should be noted that the hyperoxidized fraction of total 2-CysPRX pool along the circadian rhythm was small, and partly insignificant in animal cells and plants [64]. Therefore, the significance of this finding, and the question of gain or loss of function of the hyperoxidized form remains to be studied [65]. Nevertheless, it was suggested for animal cells that mitochondria play a major role in determining the circadian redox dynamics, with a particular role of the animal 2-CysPRX PRXIII and sulfiredoxin [66].

## 4. The Protective Function of PRXIIF in Seeds

The mitochondrial PRXIIF (the equivalent to human PRX5) belongs to the second group of peroxiredoxins, namely the atypical 2-CysPRXs. The functional unit of PRXIIF is the monomer, since the peroxidatic Cys_P_ at position C59 and the resolving Cys_R_ at position C84–numbering according to pea PRXIIF localize on the same polypeptide; however, higher molecular mass assemblies such as hexamers have been observed by size exclusion chromatography [67] and as unit cell in protein crystals [68]. PRXIIF catalyzes the reduction of H_2_O_2_ and organic hydroperoxides with a preference for H_2_O_2_ rather than alkyl hydroperoxides. As concluded from *PRXIIF* transcript quantification, PRXIIF is a housekeeping constituent of mitochondria [58,69,70] and involved in mitochondrial redox homeostasis. PRXIIF deficiency is compensated in *Arabidopsis thaliana* under normal growth conditions, but when oxidative stress occurs, e.g., in cadmium-exposed roots, *prxIIF* mutants display a severe growth retardation [59].

The *A. thaliana* genome codes for five type II PRXs, namely, the three cytosolic PRXIIB, C, D, one plastidic PRXIIE and one mitochondrial PRXIIF [58,69]. In the model tree poplar (*Populus trichocarpa* Torr. and A. Gray ex. Hook.), four genes encode type II PRXs, namely, two cytosolic PRXII isoforms, one chloroplast PRXIIE and one mitochondrial PRXIIF [70], and in sugar beet *Beta vulgaris* L., only one gene each encodes a cytosolic PRXIIB, a plastidic PRXIIE and one mitochondrial PRXIIF [29]. With the availability of additional full-genome sequences, it appears that all photosynthetic eukaryotes contain a mitochondrial PRXIIF. In addition, plants contain at least one of each, a nuclear 1-Cys PRX, a plastidic 2-Cys PRX, and a chloroplast PRXQ.

In a study conducted on poplar leaves, PRXIIF was found to be unresponsive to any abiotic factors, such as heavy metals or cold [70], and the amount of PRXIIF in leaves remained stable. In a converse manner, PRXIIF in pea (*Pisum sativum* L.) increased after exposure to heavy metals [49]. The only factor that distinguished PRXIIF activity in poplar was aging [70], a process heavily influenced by ROS. Although the loss of half of the mitochondria in the tissues studied was observed during aging, PRXIIF levels remained unchanged [70]. Previous studies have described PRXIIF in *A. thaliana* as a protein that is involved in protecting mitochondria from damage by ROS [8]. Sweetlove et al. (2002) detected PRXIIF as polypeptide with strongly increased abundance in mitochondria isolated from H_2_O_2_- or menadione-treated *A. thaliana* cell culture [16]. This analysis also demonstrated the severe damage to mitochondrial functions upon oxidative stress.

As outlined above, the combination of low antioxidant activity and ROS production is considered as a main determinant of decreasing seed viability [15], since ROS cause oxidative damage and metabolic dysfunction, disrupt membrane systems, and cause oxidative damage to mitochondrial proteins, lipids, and DNA [71].

H_2_O_2_ strongly affects disulfide bond formation and the correct tertiary structure of the protein [9]. The loss of germination capacity in long-term stored beech seeds *Fagus sylvatica* L. (in which ROS levels increased) was associated with increasing ROS levels such as superoxide radical (O_2_^−●^), H_2_O_2_ and products of lipid hydroperoxide (LHPOs) [72].

Studies on seeds of *Acer* spp. and *Fagus sylvatica* have shown that changes in PRXIIF correlate with altered redox reactions in mitochondria [73,74], which are essential for maintaining high seed viability. This is in line with observations in *A. thaliana*, where PRXIIF protects stored seeds from oxidative stress [59,75] and may serve as a marker of oxidative stress in leaves [16,49]. This fact has a significant effect on seed aging, which can be confirmed by elevated PRXIIF levels in stored beech seeds [74]. The marked changes in the redox state that occur during seed storage lead to a reduced seed viability and thus to an acceleration of the aging process. A decline in antioxidant capacity opens the way for processes associated with programmed cell death, which is involved in seed aging [71]. Some of the most important factors influencing this process are the changes occurring in mitochondria, as the energy center of the cell [15,76,77].

Apparently, mitochondrial PRXIIF contributes to the maintenance of redox homeostasis and thus affects delaying seed aging. How significant this impact is and whether we could control it is still unknown. Seed aging is a complex molecular, biochemical, and physiological process. Embryos are susceptible to oxidative stress, so seed viability is reduced during storage. Studies on the in situ localization of ROS show that, during storage for several years (8–11 years), the fluorescent signal for O_2_^−^ and H_2_O_2_ is more intense in the apical meristem of the embryo than in the seeds [74]. However, longer storage (13 years) leads to an intense fluorescence signal for seeds [74].

High levels of ROS in the meristematic zone of embryonic axes probably accelerate seed aging and inhibit germination during long-term storage. ROS modify the thiol redox potential in the cell by changing the redox ratio of reduced glutathione (GSH) to oxidized glutathione (GSSG) [78], and maintaining redox balance in seed cells has a major impact on upholding seed viability during drying and long-term storage [13].

Glutathione (γ-Glu-Cys-Gly) is the most abundant low molecular mass thiol compound in cells. It plays a decisive role during disulfide bond formation. In contrast, oxidized glutathione (GSSG) acts as an oxidant in the formation of disulfide bonds in proteins, primarily by *S*-glutathionylation. Reduced glutathione (GSH) also assists in cleaving improperly bridged disulfide bonds [9]. The interfering biochemical processes involved in seed dormancy are quite complex, and the redox regulatory network plays a major role. Answering the question about the role of PRXIIF will not be simple. In addition to studying the aging process of seeds in general, we need to distinguish two types of seeds: orthodox and recalcitrant. PRXIIF amounts and activity change slightly differently in the two seed types. During progressing desiccation of seeds, PRXIIF behaved differently at the level of *PRXIIF* transcript, PRXIIF protein and at the level of posttranslational modifications between *Acer platanoides* L. (orthodox seeds) and *Acer pseudoplatanus* L. (recalcitrant seeds) [73]. Redox regulation differs at the mitochondrial level in both species, and PRXIIF during seed drying and desiccation defines physiological differences between orthodox and recalcitrant seed types of Norway maple and sycamore during [13].

Therefore, further research should focus on differentiating the seed types. More detailed studies are needed to accurately determine the relevance to the aging process and the function of PRXIIF during oxidative stress.

## 5. Conclusions

PRXIIF and the thioredoxins TRX-o and TRX-h, but also other elements of the mitochondrial redox regulatory network such as glutathione reductase, are important in maintaining the proper redox state of plant mitochondria. The redox balance in mitochondria affects their functioning. Adjusting these parameters likely can improve the resistance of the seed to oxidative stress during storage and reduce cell damage, thus delaying the initiation of the aging process. Understanding the signals responsible for the redox regulatory state and the involvement of PRXIIF and TRX-h and TRX-o in seed mitochondria can bring us closer to understanding the causes of the aging process of seeds during storage. If we would establish an impact of PRXIIF in the future, we might also have an effect on maintaining the viability of stored seeds, or we might be able to predict the behavior of seeds. Recent work on *A. thaliana* using dynamic cell imaging probes and redox proteomics approaches combined with mutants lacking TRX-o1, glutathione reductase, NADPH-dependent thioredoxin reductase a/b compared to wildtype demonstrated the significance of redox switching from a more oxidized to a more reduced state in general and the importance of these proteins in particular [79]. Similar work is needed for dissecting the role of PRXIIF and GPXL6 in seed germination, first in model plants such as *A. thaliana*, but then in seeds of crop plants and trees differing in seed storage behavior.
